# Trends in suicide in a Lithuanian urban population over the period 1984–2003

**DOI:** 10.1186/1471-2458-6-184

**Published:** 2006-07-13

**Authors:** Abdonas Tamosiunas, Regina Reklaitiene, Dalia Virviciute, Diana Sopagiene

**Affiliations:** 1 Department of Population Studies, Institute of Cardiology, Kaunas University of Medicine, Kaunas, Lithuania

## Abstract

**Background:**

Throughout the last decade of the twentieth century, Lithuania had the highest suicide rates in Europe among both men and women aged 25–64 years. The rates increased from 1986 until 1995, but later there was a slight decrease. This paper describes the trends in suicide deaths in urban population in Lithuania by gender, dates and suicide method over the period 1984–2003.

**Methods:**

Data from the regional mortality register were used to analyze suicide deaths among all men and women aged 25–64 years in Kaunas city, Lithuania over the period 1984–2003. Age-standardized death rates per 100,000 persons (using European standard population) were calculated by gender, suicide method and dates. A joinpoint regression method was used to estimate annual percentage changes (EPACs) and to detect points where the trends changed significantly.

**Results:**

The frequency of death by suicide among males was 48% higher in 1994–2003 than in 1984–1993. The corresponding increase among females was 28%. The most common methods of suicide among men were hanging, strangulation and suffocation (87.4% among all suicide deaths). The proportions of hanging, strangulation and suffocation in males increased by 6.9% – from 83.9% to 89.7% – compared to a 24.2% increase in deaths from handgun, rifle and shotgun firearm discharges and a 216.7% increase in deaths from poisoning with solvents, gases, pesticides and vapors. Among females, the most common methods of suicide were hanging, strangulation and suffocation (68.3% of all suicide deaths). The proportion of hanging deaths among females increased during the time period examined, whereas the proportion of poisonings with solid or liquid substances decreased.

**Conclusion:**

Suicide rates increased significantly among urban men aged 25–64 years in Lithuania throughout the period 1984–2003, whereas among women an increasing but statistically insignificant trend was observed. There were changes in the suicide methods used by both men and women. Changes in the choice of method may have contributed to the changes in suicide rates.

## Background

Surveillance of mortality trends has a long and continuing tradition in health research and has generated many useful insights into the determinants of disease and causes of death. Suicide is an important and preventable public health problem and a considerable drain on resources in both primary and secondary health care settings.

Compared to adjacent countries and other European states, Lithuania had the highest suicide rates among both men and women aged 25–64 years throughout the last decade of the twentieth century [1]. The suicide rates among Lithuanian men increased from 1986 until 1995, then a decreasing trend ensued. Similar but less clear and marked changes in suicide trends were detected among Lithuanian women.

A number of studies have analyzed the relationship between suicide rates and health and socio-economic factors such as gender, urban-rural differences, unemployment rates and others [2,3]. Identifying time trends and methods of suicide by different genders and in different regions of the specific country are important tasks for researchers and public health officials seeking to develop intervention strategies and comprehensive suicide prevention activities.

The aim of this study was to identify and compare the methods of suicide among urban Lithuanian middle-aged males and females, and to examine trends in suicide deaths in the urban population over the past two decades.

## Methods

Kaunas is the second largest city of Lithuania, a former socialist country in transition to a market economy, with an area of 157 km^2 ^and a population of about 360,000. Kaunas, an industrial city with emphasis on textiles, manufacturing and the food industry, is located in the center of the state. More than 90% of the inhabitants of Kaunas are ethnic Lithuanians.

Kaunas city ischemic heart disease and mortality registers were started in 1971, so we could analyze long term trends in mortality rates in the city population. Data from the regional (Kaunas city) mortality register were used to analyze suicide deaths among all men and women in the city aged 25–64 years. Deaths registered during 1984–2003 were included if the cause of death was recorded as suicide (ICD-9 E950–E959 or ICD-10 X60–X84). We checked all suicide cases during the study period and found no cases classified as undetermined intent deaths, so we did not include undetermined intent deaths in our analysis. Deaths of persons who lived outside the city boundaries but died in Kaunas were excluded so that the appropriate city demographic data could be used.

We analyzed suicide deaths over two different time periods: the economically fairly stable period before and few years after the regaining of Lithuanian independence (1984–1993), and the period 1994–2003, when the population started to adopt new social, political and economic changes.

Population figures were taken from the annual reports of the Central Statistical Department of Kaunas city. Age-standardized death rates per 100,000 persons (using European standard population) were calculated for men and women aged 25–64 using ten-year age groups. Trends in age-standardized suicide rates were calculated by joinpoint regression using Joinpoint software version 2.6 [4]. The joinpoint regression was adjusted to estimate annual percentage changes (EPAC) and to detect points where the trends changed significantly [5]. The number of joinpoints was determined by performing several permutation tests, each of which had a correct asymptotic significance level. These p-values were found using Monte Carlo methods and applying Bonferroni corrections. For each EPAC estimate, the corresponding 95% confidence interval (CI) was calculated. The number of joinpoints was limited to a maximum of three.

All data other than joinpoints were analyzed using Excel and SPSS software packages. A significant difference between the parameters was considered to be greater than 1.95 (p < 0.05).

The investigation conformed to the principles outlined in the Declaration of Helsinki and was approved by the regional ethics committee.

## Results

There were 1437 deaths recorded as suicides during the time period investigated. Of these deaths, 81.6% occurred in males (n = 1172) and 18.4% in females (n = 265).

### Gender and method of suicide

Table 1 shows the distribution of suicide deaths by method and gender during the period 1984–2003. The most common methods of suicide among persons aged 25–64 years in the Kaunas population were hanging, strangulation and suffocation, poisoning with solid or liquid substances, handgun, rifle, shotgun, larger firearm or unspecified firearm discharges, and jumping from high places. Hanging, strangulation and suffocation were the most common methods among both men and women. They accounted for a significantly greater proportion of suicide deaths among males than among females. A greater proportion of males than females used handgun, rifle, shotgun, larger firearm or unspecified firearm discharges as the method of suicide. Poisoning with solid or liquid substances, drowning and submersion and jumping from high places accounted for a significantly greater proportion of female than male suicide deaths.

### Time period and method of suicide

When changes over time in suicide methods among males were examined (Table 2), increases in the proportions of poisoning with solvents, gases, pesticides and vapors (216.7%), hanging, strangulation and suffocation (6.9%), and handgun, rifle, shotgun, larger firearm or unspecified firearm discharges (24.2%) were apparent during the period 1994–2003. There were decreases in the proportions of poisoning with solid or liquid substances (84.2%), jumping from high places (73.7%), self-harm by jumping or lying before a moving object, crashing a motor vehicle or other means (84.2%) and drowning and submersion (25.0%). There was an overall 48.2% increase in the rate of male suicides between 1984–1993 and 1994–2003.

Among women (Table 3), there were increases in the proportions of poisoning with solvents, gases, pesticides and vapors (422.2%), hanging, strangulation and suffocation (30.4%) and drowning and submersion (252.9%). Decreases were observed in the proportions of poisoning with solid or liquid substances (73.5%), jumping from high places (42.9%), self-harm by jumping or lying before a moving object or crashing a motor vehicle or other means (100%), and self-harm by smoke, fire, flames, steam, hot vapors, and hot and sharp objects (37.2%). There was an overall 27.7% increase in the rate of female suicides between 1984–1993 and 1994–2003.

Mortality from suicide averaged 5.3 times higher in males than in females – 61.3 vs. 11.5 per 100,000 – throughout the period of study (1984–2003). The difference in mortality rates between men and women increased from 4.9-fold in 1984–1993 to 5.7-fold in 1994–2003.

### Age group, gender and time period

In males, suicide rates ranged from 30.7 per 100,000 in the 25–34 age group to 54.0 per 100,000 in the 55–64 age group (p < 0.001) during 1983–1993, and from 41.0 per 100,000 to 84.8 per 100,000 (p < 0.001), respectively, during 1994–2003. Suicide rates in all four age groups examined rose between 1984–1993 and 1994–2003. The highest suicide rates in each time period were in the 45–54 age group. Among females, it was only in this age group that an increase in suicide rate was detected between 1984–1993 and 1994–2003. The highest rates were registered in the 55–64 age group in 1984–1993 (15.7 per 100,000) and in the 45–54 age group in 1994–2003 (19.0 per 100,000).

**Figure 1:**
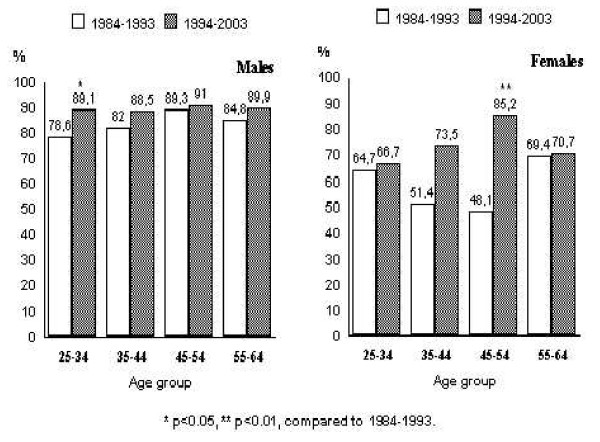
Proportions of hanging, strangulation and suffocation in Kaunas during the period of 1984–2003 by age, gender and time period.

The proportion of hanging, strangulation and suffocation among men increased in the 25–34 age group only (Figure 1) between 1984–1993 and 1994–2003. In females, only the 45–54 age group showed an increase in the proportion of suicide by these methods. The proportions of hanging, strangulation and suffocation for both males and females showed no statistically significant differences between age groups.

### Changes in mortality from suicide over time

The results of the joinpoint analysis of time trends of mortality from suicide are shown in Table 4. In males, suicide rates increased significantly throughout the period of the study. A significant increase in suicide rates – by 4.6% per annum during 1984–1997 – was succeeded by a decreasing trend, although the change was not statistically significant. In females, there was an overall increasing trend though it was not statistically significant. The decrease between 1984 and 1986 (21.2% per year) was not significant, and the subsequent increase (2.3% per year) was of borderline significance (p = 0.08).

The results of the joinpoint analysis of time trends of hanging, strangulation and suffocation are shown in Table 5. In both genders the rates of these methods of suicide increased significantly throughout the period of the study. Males showed a non-significant decrease during 1984–1993 followed by a significant increasing trend (4.7% per year, p = 0.0001). Females showed a non-significant decrease during 1984–1988 followed by a significant increasing trend (5.0% per year, p = 0.04) during 1988–2003.

The joinpoint model could not be used for other individual suicide methods owing to the small number of deaths; the requirement for at least one case of death each year during the time period analyzed could not be met.

## Discussion

This study emphasizes the already well-established fact that suicide rates in the Lithuanian population are very high, especially among men [6]. The data set of the present study is highly representative. The study period covered 20 consecutive years, making it possible to evaluate the real trends in suicide mortality for the entire regional urban population aged 25–64 years. We applied joinpoint regression to the data and quantified the observed changes. To our knowledge, this is the first analysis of suicide mortality of this type performed for the urban Lithuanian population.

The data for the study were based on information on deaths during the period 1984–2003, where suicide was recorded as an underlying cause, and were obtained from death certificates. Quantitative and qualitative analysis during the period 1970–1990 demonstrated that suicide mortality statistics were reliable for Lithuania, Estonia and Latvia [7].

The limitation of the study is that during the study period – in 1998 – the 9^th ^revision of the ICD was replaced by the 10^th^. This means that the second period (1994–2003) of the study was covered by two different classifications of causes of death. The change in the death registration system could have led to an artificial fluctuation in the numbers of cause-specific deaths.

This study shows that local suicide rates in Kaunas city developed in the same pattern as national statistics for the whole of Lithuania: during 1984–2003, suicide rates among male inhabitants of Kaunas city showed a significantly increasing trend [6,8]. The rate among women was 27.7% higher during 1994–2003 than during 1984–1993. Over the whole study period, suicide rates increased by 1.3% annually, p > 0.05. Similar increases in suicide mortality were observed over the whole country and among regional male and female populations during the 1990s in several European countries including Bulgaria, Latvia, the Russian Federation, Spain and Ukraine [6,9]. Most countries exhibited a decline in suicide mortality among both men and women over the same period, but in some cases, men and women showed opposite trends. Our data also confirmed earlier observations that the suicide rate among males is at least three- or four-fold higher than among females [10-12].

Variations in suicide rates can be attributable to many different influences. Personal and social and economic factors, especially unemployment rates, have most often been investigated [13,14]. Changes in suicide rates could be attributed to changes in alcohol consumption. In Finland, both the rapid increase in mortality from alcohol-related causes and a simultaneous increase in mortality from accidents and suicides was largely caused by the rapid increase in alcohol consumption during the 1980s [15]. In Kaunas over the period 1984–2003, there were increases in both the proportion of drinkers aged 35–64 years who consumed alcohol very frequently – several times a week or every day – and the amount of alcohol consumed (personal unpublished data). The results of the international study by FINBALT Health Monitor showed similar data for the whole of Lithuania during the last decade [16,17].

Our findings indicate that the increase in suicide rates coincided with marked changes in the pattern of methods. There was a rise in the rate of hanging, strangulation and suffocation among both men and women in Kaunas. Hanging is an easy and reliably lethal method that is most common in many countries, especially among men [11,18,19]. Firearms have the highest case fatality rates, followed by drowning and hanging [20]. The most striking changes in male suicide methods were marked increases in deaths from poisoning with solvents, gases, pesticides, vapors, and deaths from handgun, rifle, shotgun, larger firearm or unspecified firearm discharges. We suggest that the increase in these rates could be related to changes in Lithuanian law enabling easier access to firearms – it is possible for each citizen to obtain a handgun legally for self-protection.

## Conclusion

Suicide rates increased significantly among urban men aged 25–64 years in Lithuania throughout the period 1984–2003. Females showed a non-significant increasing trend. There were changes in the methods used by both men and women. Changes in the choice of suicide method may have contributed to the changes in rates. It is important to understand the reasons for this to allow appropriate intervention strategies to be considered.

## Conflict of interests

The author(s) declare that they have no conflicts of interests.

## Authors' contributions

AT had the idea for the study, contributed to the design and the interpretation, and drafted the paper. Regina Reklaitiene contributed to the design and commented on the interpretation of the results. Dalia Virviciute and Diana Sopagiene analyzed the data. All authors read and approved the final draft of the paper.

## Pre-publication history

The pre-publication history for this paper can be accessed here:



## Figures and Tables

**Table 1:** Methods of suicide in Kaunas population by gender during the period of 1984–2003

**Primary cause and ICD codes**			**Males**		**Females**		**Total**	
**Primary cause**	**ICD-9**	**ICD-10**	**No of deaths**	**%**	**No of deaths**	**%**	**No of deaths**	**%**

Poisoning with solid or liquid substances	E950	X60–65	22	1.9	35	13.2***	57	4.0
Poisoning with solvents, gases, pesticides and vapours	E951–952	X66–69	16	1.4	8	3.0	24	1.7
Hanging, strangulation and suffocation	E953	X70	1024	87.4	181	68.3***	1205	83.9
Drowning and Submersion	E954	X71	8	.7	11	4.2**	19	1.3
								
Handgun, rifle, shotgun, larger firearm or unspecified firearm discharge	E955	X72–75	44	3.8	1	.4***	45	3.1
Self-harm by smoke, fire, flames, steam, hot vapours, and hot and sharp objects	E956	X76–79	22	1.9	9	3.4	31	2.2
Jumping from a high places	E957	X80	25	2.1	14	5.3*	39	2.7
Self-harm by jumping or lying before a moving object, crashing of a motor vehicle or other means	E958–959	X81–84	11	.9	6	2.3	17	1.2
Total	E950–959	X60–84	1172	100.0	265	100.0	1437	100.0

Statistically significant as compared to males – *p < .05, **p < .01, ***p < .001.

**Table 2:** Methods of suicide in males in Kaunas during the period of 1984–2003 by time period

**Primary cause and ICD codes**			**1984–1993**		**1994–2003**			**1984–2003**	
		
**Primary cause**	**ICD-9**	**ICD-10**	**No of deaths**	**%**^**1**^	**No of deaths**	**%**^**1**^	**%**^**2**^	**No of deaths**	**%**^**1**^
Poisoning with solid or liquid substances	E950	X60–65	18	3.8	4	.6	-84.2	22	1.9
Poisoning with solvents, gases, pesticides and vapours	E951–952	X66–69	3	.6	13	1.9	216.7	16	1.4
Hanging, strangulation and suffocation	E953	X70	398	83.9	626	89.7	6.9	1024	87.4
Drowning and submersion	E954	X71	4	.8	4	.6	-2.5	8	.7
Handgun, rifle, shotgun, larger firearm or unspecified firearm discharge	E955	X72–75	15	3.3	29	4.1	24.2	44	3.7
Self-harm by smoke, fire, flames, steam, hot vapours, and hot and sharp objects	E956	X76–79	9	1.9	13	1.8	-5.3	22	1.9
Jumping from a high places	E957	X80	18	3.8	7	1.0	-73.7	25	2.1
Self-harm by jumping or lying before a moving object, crashing of a motor vehicle or other means	E958–959	X81–84	9	1.9	2	.3	-84.2	11	.9
Total	E950–959	X60–84	474	100.0	698	100.0		1172	100.0

^**1**^**% – **proportion (%);

^**2**^**% – **% change from the first to the second time period;

**Table 3:** Methods of suicide in females in Kaunas during the period of 1984–2003 by time period

**Primary Cause and ICD codes**			**1984–1993**		**1994–2003**			**1984–2003**	
		
**Primary cause**	**ICD-9**	**ICD-10**	**No of deaths**	**%**^**1**^	**No of deaths**	**%**^**1**^	**%**^**2**^	**No of deaths**	**%**^**1**^
Poisoning with solid or liquid substances	E950	X60–65	26	22.6	9	6.0	-73.5	35	13.2
Poisoning with solvents, gases, pesticides and vapours	E951–952	X66–69	1	.9	7	4.7	422.2	8	3.0
Hanging, strangulation and suffocation	E953	X70	67	58.3	114	76.0	30.4	181	68.3
Drowning and submersion	E954	X71	2	1.7	9	6.0	252.9	11	4.1
									
Handgun, rifle, shotgun, larger firearm or unspecified firearm discharge	E955	X72–75	0	0	1	.6	-	1	.4
Self-harm by smoke, fire, flames, steam, hot vapours, and hot and sharp objects	E956	X76–79	5	4.3	4	2.7	-37.2	9	3.4
Jumping from a high places	E957	X80	8	7.0	6	4.0	-42.9	14	5.3
Self-harm by jumping or lying before a moving object, crashing of a motor vehicle or other means	E958–959	X81–84	6	5.2	0	0	-100.0	6	2.3
Total	E950–959	X60–84	115	100.0	150	12.9		265	100.0

^**1**^**% – **proportion (%);

^**2**^**% – **% change from the first to the second time period;

**Table 4:** Trends in age-standardized suicide rates in Kaunas men and women aged 25–64 years during 1984 to 2003

Men					Women				
Joinpoints (Years)	Time period	EPAC^1^	95% CI^2^	p-value	Joinpoints (Years)	Time Period	EPAC^1^	95% CI^2^	p-value

0 jointpoints	1984 – 2003	+3.4	+2.1 – +4.7	.0003	0 jointpoints	1984 – 2003	+1.2	-.8 – +3.3	.2
1997	1984 – 1997	+4.6	+2.1 – +7.3	.0014	1986	1984 – 1986	-21.2	-62.5 – +65.9	.5
	1997 – 2003	-.2	-7.9 – +8.2	.96		1986 – 2003	+2.3	-.3 – +4.9	.08
1990	1984 – 1990	-3.7	-8.8 – +1.6	.15					
1993	1990 – 1993	+20.6	-12.2 – +65.7	.22					
	1993 – 2003	+.5	-1.9 – +3.0	.66					

^1^EPAC – estimated annual percent change;

^2^95% CI – 95% confidence intervals;

**Table 5:** Trends in age-standardized rates of hanging, strangulation and suffocation in Kaunas men and women aged 25–64 years during 1984 to 2003

Men					Women				
Joinpoints (Years)	Time period	EPAC^1^	95% CI^2^	p-value	Joinpoints (Years)	Time period	EPAC^1^	95% CI^2^	P-value

0 jointpoints	1984 – 2003	+4.0	+2.5 – +5.6	.00002	0 jointpoints	1984 – 2003	+3.6	+.7 – +6.6	.02
1986	1984 – 1986	-10.4	-48.2 – +55.2	.68	1988	1984 – 1988	-5.6	-33.5 – +34.2	.7
	1986 – 2003	+4.7	+2.7 – +6.7	.0001		1988 – 2003	+5.0	+.2 – +10.1	.04
1990	1984 – 1990	-4.0	-10.2 – +2.7	.22	1992	1984 – 1992	-4.6	-13.0 – +4.6	.28
1993	1990 – 1993	+22.5	-17.8 – +82.5	.29	1995	1992 – 1995	34.3	-42.4 – +213.0	.47
	1993 – 2003	+1.1	-2.0 – +4.3	.46		1995 – 2003	-3.1	-11.6 – +6.3	.48

^1^EPAC – estimated annual percent change;

^2^95% CI – 95% confidence intervals;

## References

[B1] World Health Organization/Europe (2005). European Mortality Database (MDB) HFA. http://www.who.dk/InformationSources/Data/20011017_1.

[B2] Schapira K, Linsley KR, Linsley A, Kelly TP, Kay DWK (2001). Relationship of suicide rates to social factors and availability of lethal methods. Comparison of suicide in Newcastle upon Tyne 1961–1965 and 1985–1994. Br J Psych.

[B3] Qin P, Agerbo E, Westergard-Nielsen N, Eriksson T, Mortensen PB (2000). Gender differences in risk factors for suicide in Denmark. Br J Psych.

[B4] National Cancer Institute (2002). Joinpoint Regression Program Version 2.6. http://srab.cancer.gov/joinpoint/download.html.

[B5] Kim HJ, Fay MP, Feuer EJ, Midthune DN (2000). Permutation tests for joinpoint regression with application to cancer rates. Stat Med.

[B6] Levi F, Vecchia C, Lucchini F, Negri E, Saxena S, Maulik PK (2003). Trends in mortality from suicide, 1965–99. Acta Psychiatr Sacnd.

[B7] Wasserman D, Varnik A (1998). Reliability of statistics on violent death and suicide in the former USSR, 1970–1990. Acta Psychiatr Scand.

[B8] Kalediene R, Petrauskiene J (2004). Inequalities in daily variations of deaths from suicide in Lithuania: identification of possible risk factors. Suicide Life Threat Behav.

[B9] Webster P (2003). Suicide rates in Russia on the increase. Lancet.

[B10] Canetto SS, Sakinofsky I (1998). The gender paradox in suicide. Suicide Life Threat Behav.

[B11] Stark C, Hopkins P, Gibbs D, Rapson T, Belbin A, Hay A (2004). Trends in suicide Scotland 1981 – 1999: age, method and geography. BMC Public Health.

[B12] Gunnell D, Middleton N (2003). National suicide rates as an indicator of the effect of suicide on premature mortality. Lancet.

[B13] Hawton K, Harriss L, Hodder K, Simkin S, Gunnell D (2001). The influence of the economic and social environment on deliberate self harm and suicide: an ecological and person-based study. Psychol Med.

[B14] Blakely TA, Collings SC, Atkinson J (2003). Unemployment and suicide. Evidence for a causal association?. J Epidemiol Community Health.

[B15] Valkonen T, Martikainen P, Jalovaara M, Koskinen S, Martelin T, Makela P (2000). Changes in economic inequalities in mortality during an economic boom and recession among middle-aged men and women in Finland. Eur J Public Health.

[B16] Grabauskas V, Klumbiene J, Petkeviciene J, Dregval L, Cepaitis Z, Nedzelskiene I (1995). Health behaviour among Lithuanian adult population, 1994.

[B17] Grabauskas V, Klumbiene J, Petkeviciene J, Dregval L, Saferis V, Prattala R (2001). Health behaviour among Lithuanian adult population, 2000.

[B18] Abe R, Shiori T, Nishimura A, Nushida H, Ueno Y, Kojima M (2004). Economic slump and suicide method: Preliminary study in Kobe. Psychiatri Clin Neurosci.

[B19] Pesonen TM, Tacke U, Karkola KI, Hintikka, Lehtonen J (2004). Gender-related changes in suicide rates and methods in Eastern Finland from 1988 to 1997. Nord J Psychiatry.

[B20] Shenassa ED, Catlin SN, Buka SL (2003). Lethality of firearms relative to other suicide methods: a population based study. J Epidemiol Community Health.

